# A Mild Case of Cerebro-Spinal Meningitis, Followed by Blindness

**Published:** 1874-07

**Authors:** Charles Shaffner


### A Mild Case of Cerebro-Spinal Meningitis followed by Blindness.

By Charles Shaffner, M. D.

The following case is of interest on account of the unexpected and unfortunate sequelæ:

I was called on March 16 to visit Eugene H., a bright, chubby little boy, six years old, and apparently of good constitution, whom I found suffering with headache, tongue coated with a whitish fur, constipation, tendency to nausea, and high fever. He had been complaining with these symptoms for about four days, and soon grew worse, with severe occipital headache, rather marked retraction of the head, tendency to internal strabismus, which was fully developed in a week, but lasted only a few days, with equally dilated pupils; coat on tongue turned brown, no sordes, rather obstinate constipation, and tendency to nausea. I always found his lower extremeties flexed. He had subsultus tendinum, slight pains in arms and legs, but no convulsion, was very restless, sleepless, and slightly delirious at night, but in daytime was crabbed, and only desired to be let alone.

In two weeks he was convalescent, when we discovered that he was almost blind; he said, "I lost my eyes when I was sick." He then complained of pain over his eyes, and this, with some dilatation of the pupils, was all that called attention to the eyes.

He is now in full convalescence, but has only slight light-perception in both eyes.

Dr. Strawbridge examined the eye-ground, and found "choked disk," giving a diagnosis of impending atrophy of the nerve.

Dr. J. Lewis Smith, of New York, in his article on "Cerebro-Spinal Fever," in *Amer. Jour. Med. Science*, for October, 1873, mentions, as sequelæ, strabismus, conjunctivitis, ulcer of cornea, and cataractous lens. There has never been any conjunctivitis, iritis, or keratitis in my case, and at present the media are clear, but the pupils still dilated. The hearing was also very imperfect for a few days.

The treatment was chiefly bromide of potassium and quinine, with citrate of potassium as a febrifuge. Calomel and rhubarb were used to relieve constipation. Sinapisms were placed on the extremities. He is now taking cinchona and iron, with cod-liver oil.

Dr. Albert G. Heyl made a careful ophthalmoscopic examination, and has made the following report: "On account of the age of the child, it was impossible to obtain a correct record of the amount of vision, or properly to estimate the extent of the paralysis affecting the muscular apparatus of the eye; the former, judging from the movements of the child, was considerably impaired.

"There was no ptosis. Both recti externi were paralyzed, especially the left, giving rise to marked internal strabismus on that side. The third nerve on the left side was also implicated, as was evidenced by the left cornea occupying a higher position than the right. Both pupils were widely dilated. Media perfectly clear. In the right eye hypermetropia $\frac{1}{22}$, in the left hypermetropia $\frac{1}{31}$, existed. The fundus of each eye presented a marked example of the 'choked disk;' the distinct swelling of the papilla, its clouded, reddish appearance, the radiating stripes along the course of the nerve-facicles, and the dilated and tortuous retinal veins, were all sufficiently pronounced in character to render the diagnosis easy. There were no ecchymoses. The apex of the left papilla was best defined with a + 10; that of the right, with a + 12."—*Philadelphia Medical Times.*

